# Improving Diagnostic Yield for Analyzing Periodic Electrograms in the Remote Detection of Pacemaker Lead Issues

**DOI:** 10.3390/s25030656

**Published:** 2025-01-23

**Authors:** Clement Quinonero, Marc Strik, Pierre Antoine Catalan, Pierre Mondoly, Julien Laborderie, Michel Haïssaguerre, Romain Eschalier, Pierre Bordachar, Sylvain Ploux

**Affiliations:** 1 CRCTB (Centre de Recherche Cardio-Thoracique de Bordeaux), INSERM (Institut National de la Santé et de la Recherche Médicale), University Bordeaux, U 1045, IHU Liryc, 33000 Bordeaux, France; cquinonero@chu-clermontferrand.fr (C.Q.); pierre.bordachar@chu-bordeaux.fr (P.B.); sylvain.ploux@chu-bordeaux.fr (S.P.); 2 INSERM, Service de Cardiologie-Electrophysiologie et Stimulation Cardiaque, CHU de Bordeaux, U 1045, 33000 Bordeaux, France; 3 Cardiology Department, CHU Clermont-Ferrand, 63000 Clermont-Ferrand, France; 4 Department of Cardiology, CHU de Toulouse, 31000 Toulouse, France; 5 Cardiology Department, CH de la Côte Basque, 64100 Bayonne, France

**Keywords:** pacemaker, remote, electrocardiogram, algorithm, lead dysfunction

## Abstract

Remote monitoring of pacemakers decreases patient complications and reduces public health expenses. The transmission of passive real-time electrograms (EGM) has been shown to increase the diagnostic yield, but this may add to the work burden. Passive EGMs provide snapshots without adjustments, while active EGMs modify pacemaker settings temporarily to encourage sensing and pacing, potentially revealing issues such as undersensing, oversensing, or loss of capture. The added value of active EGMs compared to the passive EGM remains to be shown. The objective of this multicenter observational study is to evaluate, in a large population of patients implanted with a pacemaker capable of transmitting both passive and active periodic EGMs, the added benefit of active periodic EGMs on diagnostic yield of pacemaker-related anomalies. In a retrospective analysis of 7068 EGMs from 2733 patients, active modes detected significantly more anomalies (6.7%) than passive alone (3.3%, *p* < 0.001), particularly for atrial leads. However, the extended duration of active EGMs (36 s versus 12 s) was the primary contributor to improved detection rates rather than the active pacing modes themselves. Our findings suggest that focusing on longer passive EGMs may enhance diagnostic yield, reducing the need for active pacing adjustments.

## 1. Introduction

The number of pacemaker implantations is increasing steadily [1,2,3]. This can be explained by the aging population in developed countries and the growing access to interventional techniques in developing countries [4]. Advancements in pacemaker technology have also contributed to this trend by broadening the range of clinical indications for implantation [5]. This has led to challenges in monitoring these patients due to the ever-increasing number of in-person consultations for device follow-up [6]. It is in this context, remote monitoring has developed over the last decade with the aim of improving patient safety and reducing the frequency of in-person consultations [7,8]. The adoption of remote monitoring has been particularly transformative in optimizing healthcare resource allocation and addressing the growing patient burden on clinics. Following favorable results in terms of morbidity [9,10,11,12], mortality [13,14], and reduction of public health expenses [15,16], cardiac societies across the globe now recommend the use of remote monitoring in all patients with a pacemaker with a class I indication [17]. Planned remote interrogation and unplanned analysis of transmitted alerts are gradually substituting in-office visits while improving the level of follow-up [18,19,20]. Despite these advancements, knowledge gaps remain regarding the diagnostic capabilities of remote technologies, particularly in identifying nuanced device-related issues [21,22].

Periodic electrograms (EGM) are, as their name implies, periodic snapshots of the atrial and/or ventricular signals automatically acquired by the implanted device. Since they are not triggered by an algorithm, they are not supposed have a high diagnostic yield. On occasion, however, these EGMs provide a unique chance to detect device malfunctions such as undersensing, oversensing, or loss of capture. Their importance lies in their ability to identify potentially serious device issues before they result in clinical symptoms or complications. The remote monitoring system of Biotronik (Home Monitoring^®^, Biotronik SE and Co. KG, Berlin, Germany) enables daily transmissions of alerts and pacemaker measurements at a set time during the night [7]. Every three months (default setting), periodic electrograms (EGM) are registered and transmitted with the goal of optimizing planned remote follow-up. The periodic EGMs from this company include not only a 12-second “passive” EGM, recorded at the programmed device settings, but also two additional strips of active EGMs: encouraged sensing and encouraged pacing, lasting 12 seconds each [23]. This approach attempts to mimic the comprehensive diagnostics traditionally performed during in-person follow-ups, ensuring a more thorough evaluation of device function. To favor atrial and ventricular sensing, the lower rate is slowed, with an extension of the atrioventricular (AV) delay. To verify atrial and ventricular capture, the lower rate is increased, with a shortening of the AV delay. These temporary and automatic parameter changes attempt to reproduce the maneuvers performed during in-office visits, which aim to unmask lead dysfunction by revealing issues of undersensing, oversensing, and loss of capture. While the benefits of transmitting passive periodic EGMs (without modification of parameters) have been demonstrated [24], the added yield of the so-called “active” EGMs remains to be shown. Quantifying this diagnostic yield could pave the way for further refinement of remote monitoring protocols and alert management systems.

The objective of this multicenter observational study is therefore to evaluate, in a large population of patients implanted with a pacemaker and remotely monitored, the diagnostic yield of analyzing periodic EGMs and to compare the rate of abnormalities detected with active periodic EGMs versus passive periodic EGMs.

## 2. Materials and Methods

### 2.1. Periodic EGM

**Figure 1:**
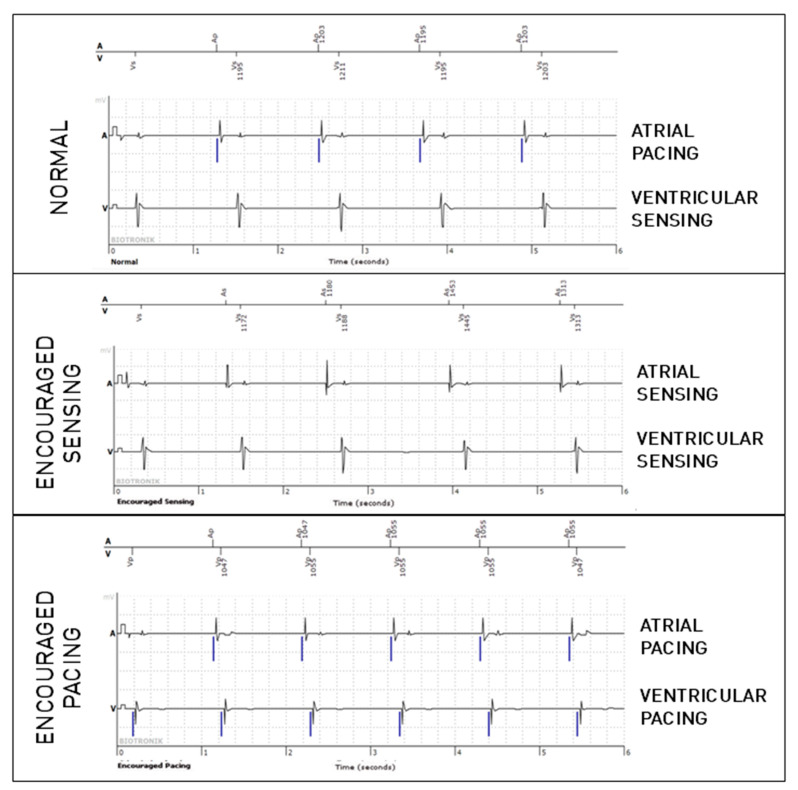
Periodic EGM showing the three modes (normal, which is passive, and the active modes, Encouraged Sensing and Encouraged Pacing) in a patient implanted because of sinus node dysfunction. In the present figure, only six-second extracts are shown for each mode (which is half of the complete tracing).

The periodic EGMs analyzed in this study are 36-second registrations consisting of three 12-second parts. The first part consists of an EGM in normal operation without programming changes (passive mode). The second part is registered during “Encouraged Sensing”, lowering the pacing rate and lengthening the atrioventricular (AV) delay according to the programmed lower rate and AV delay hysteresis, enabling the evaluation of sensing. If no hysteresis is programmed, the lower rate is decreased by 10 beats per minute, and the AV delay is lengthened by 70 ms to allow the spontaneous rhythm to appear. The third part corresponds to the “Encouraged Pacing” mode. The device increases its pacing rate by 12.5% and reprograms a short AV delay of 100 ms to favor pacing, which makes it possible to evaluate the efficiency of capture (Figure 1). The second part (Encouraged Sensing) and third part (Encouraged Pacing) are together considered to be the “active mode”.

### 2.2. Study Protocol

We performed an observational, retrospective, multicenter study conducted in the French hospitals of Bordeaux, Clermont-Ferrand, Toulouse, and Bayonne. A total of 2733 patients implanted with a single chamber (15%), dual chamber (74%), or biventricular (11%) Biotronik pacemaker were included. A Python^®^ (Spyder 6) script was used to automatically review every patient on the remote monitoring website and export the three most recent periodic EGMs in SVG format. The dataset thus consisted of 7068 periodic EGMs ready for annotation. To facilitate the reading of this large number of EGMs, an custom algorithm was developed using Matlab^®^ 2024 to simultaneously display a trace and a dropdown menu with a list of possible diagnoses. Once the diagnosis was selected, a second dropdown menu appeared to determine in which parts of the trace the anomaly had been detected in (normal mode, Encouraged Sensing, and/or Encouraged Pacing). An electrophysiologist labeled every episode according to one of twelve available diagnoses: normal, or in the case of an anomaly for atrial or ventricular lead, one of the following abnormalities: undersensing, oversensing (further specified as far-field, lead dysfunction, or external source), and loss of capture (also for the left ventricular lead, if present). Signals generated by another chamber or lead were considered far-field, and external source was used to classify signals which originated from outside the body. In case of multiple abnormalities, the clinically most important was chosen: ventricular before atrial, and priority given to lead dysfunction. The chosen diagnosis and the modes in which it was present were then automatically exported to a spreadsheet, minimizing the risk of errors that could occur through manual entry. An important aspect to consider when comparing these modes is that the passive mode is 12 s in length, and that the active mode adds 2 × 12 s to the recording, meaning the total EGM is 36 s. When comparing passive-only mode to complete periodic EGM (passive and active modes with Encouraged Sensing and Encouraged Pacing) modes we expect to find more anomalies because we have three times the opportunity to find them based on the length of the tracing alone. The research reported in this paper adhered to the Helsinki Declaration, data were de-identified prior to analysis, and the ethics review board of Bordeaux University approved the study.

### 2.3. Statistical Analysis

Prevalence was calculated for each anomaly and compared between the passive-only mode and complete periodic EGM using paired student t-tests. A Cochran’s Q test was conducted to determine whether there were significant differences in the effectiveness of the three pacing modes (i.e., normal, Encouraged Sensing, and Encouraged Pacing) for the detection of the different lead anomalies. Two-sided, pairwise multiple comparison tests were computed to determine whether the normal mode was different from either the Encouraged Sensing or the Encouraged Pacing mode. For these comparisons, the individual alpha level was adjusted using the Bonferroni method. Categorical variables are presented as absolute numbers and percentages. Continuous variables are shown as mean ± standard deviation. Statistical analyses were performed using SPSS for Windows, version 19.0 (SPSS, Inc., Chicago, IL, USA).

## 3. Results

### 3.1. Prevalence of Anomalies and Distribution Across Modes

After analysis of all 7068 periodic EGMs from 2733 patients, anomalies were detected in 473 episodes (6.7%) using both passive and active modes. When only using the passive mode, anomalies were only identified in 232 episodes, meaning 51% would be missed without the active modes.

In total, anomalies were found in 232 episodes (3.3%) during the passive mode, 274 episodes (3.9%) during Encouraged Sensing, and in 212 episodes (3%) during Encouraged Pacing, meaning that in approximately half of the episodes, anomalies were identified in at least two modes. Table 1 shows the prevalence of the possible diagnoses for the passive mode and the two active modes. The number of anomalies found and their distribution across the modes are described in Table 1.

### 3.2. Atrial Lead

**Figure 2:**
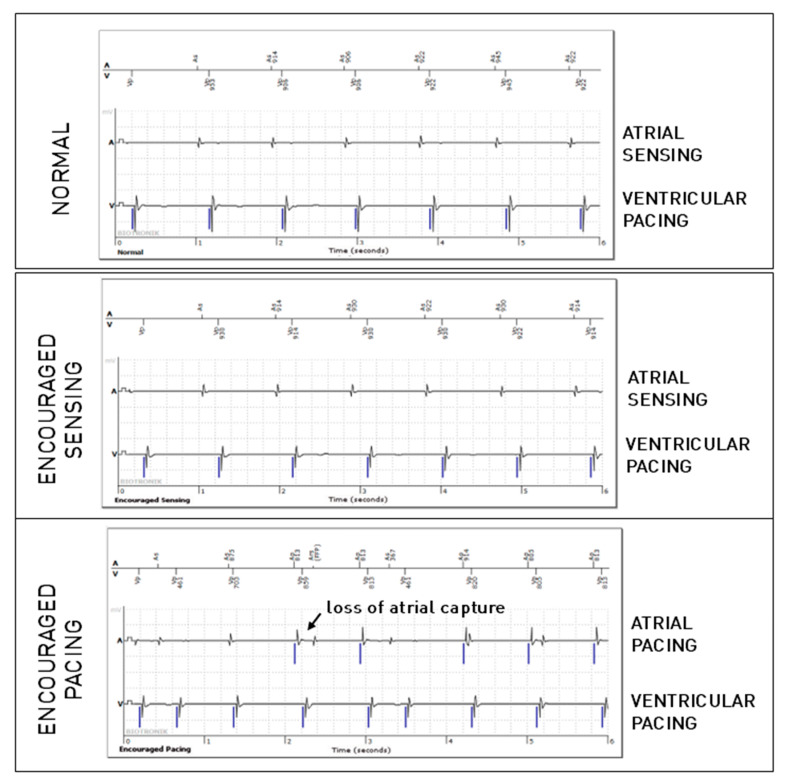
Atrial loss of capture in the “Encouraged Pacing” part of the active mode.

Out of the 5543 periodic EGMs including an atrial lead, 359 episodes revealed an atrial lead anomaly (6.5%), of which only 149 were observed during the passive mode. No significant difference was observed regarding atrial undersensing between these modes, while trends suggested more undersensing during Encouraged Sensing and less during Encouraged Pacing. Regarding far-field ventricular oversensing on the atrial lead, compared to the normal mode, significantly more anomalies were found in Encouraged Sensing (*p* < 0.001), and significantly fewer anomalies were found in Encouraged Pacing (*p* < 0.001). This can be explained by the fact that these oversensing anomalies are much more frequent with spontaneous ventricular activity than after paced ventricular activity. There were also significantly more cases of atrial oversensing due to lead dysfunction during Encouraged Pacing (*p* < 0.001). We have no certain explanation for this phenomenon, but we speculate that the tension applied to the circuit during atrial stimulation may promote the appearance of over-detected noise on the defective atrial lead. Finally, more cases of loss of atrial capture were detected during Encouraged Pacing when compared with the passive mode (*p* < 0.0001). Figure 2 shows an example of loss of atrial capture that was not visible during the passive (normal) mode, as there was no atrial pacing, but was unmasked by the active mode (Encouraged Pacing).

### 3.3. Ventricular Leads

**Figure 3:**
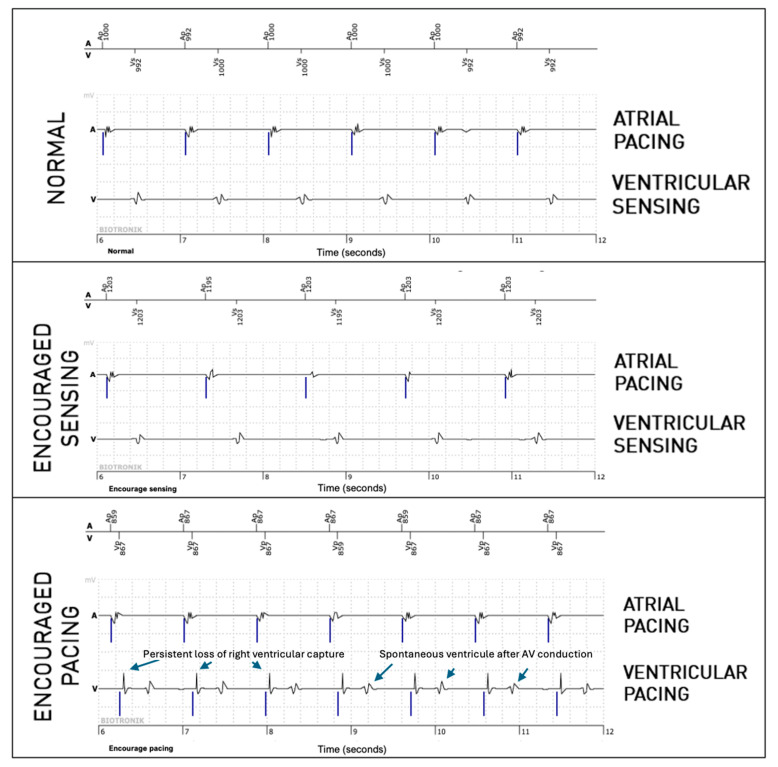
Persistent loss of right ventricular capture unmasked by the “Encouraged Pacing” part of active mode.

**Figure 4:**
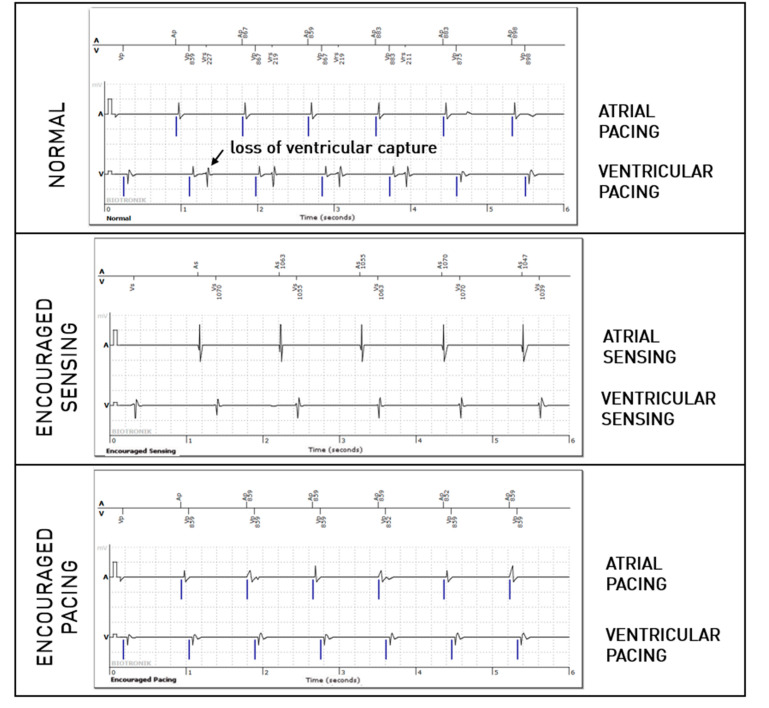
Intermittent loss of right ventricular capture during the passive “normal” mode but not during the active “Encouraged Pacing” mode.

**Figure 5:**
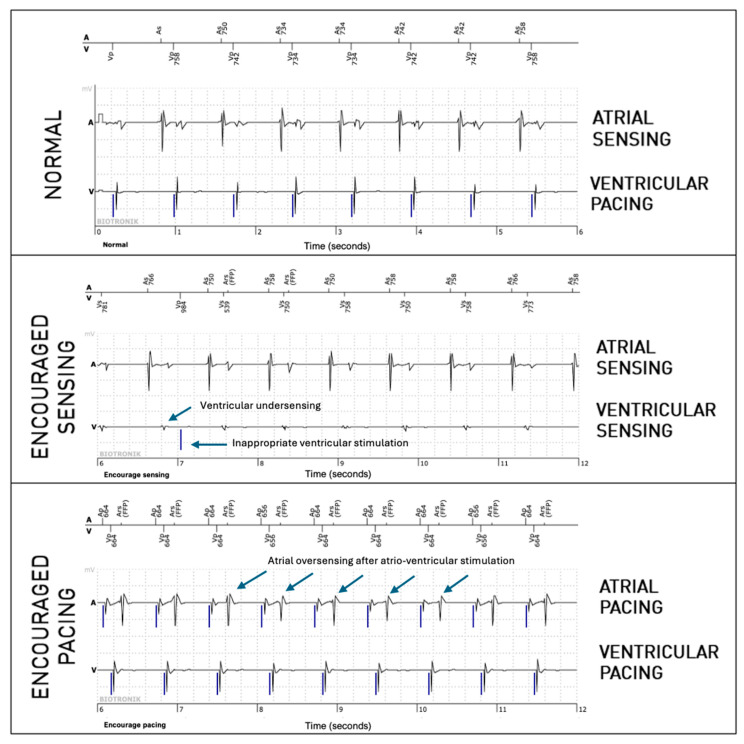
Intermittent right ventricular undersensing unmasked by the “Encouraged Sensing” part of an active mode.

**Figure 6:**
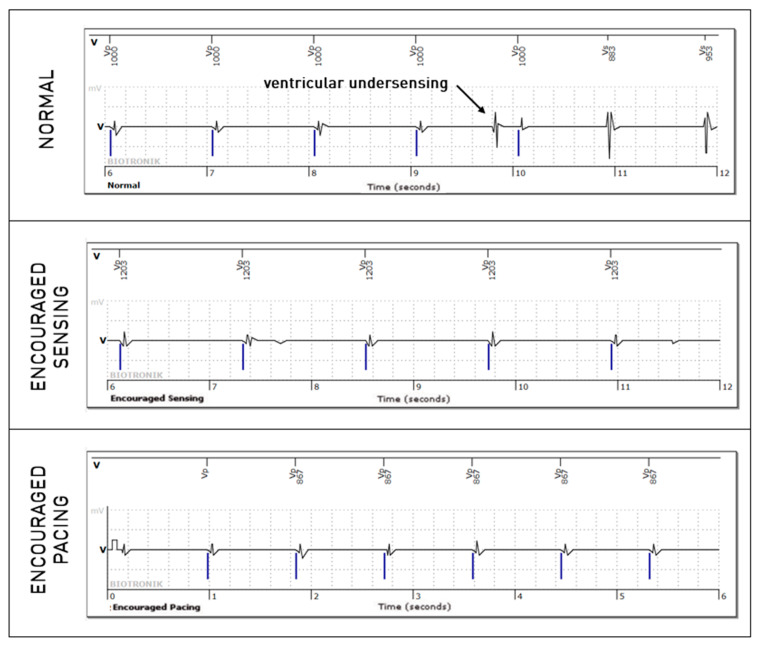
Intermittent right ventricular undersensing shown during the normal passive mode but not during the “Encouraged Sensing” part.

For the right ventricular lead, significantly more anomalies were found during the passive + active mode (67 episodes, 0.95%) than during passive-only mode (36, 0.51%, *p* = 0.008). However, this difference is mostly explained due to longer EGMs (36 s versus 12 s). Considering that anomalies could be present in multiple modes during the episode, anomalies were actually not more frequently observed in one of the three modes (58% during passive mode, 40% during Encouraged Sensing, and 45% during Encouraged Pacing, *p* = 0.15). RV undersensing issues were not seen more during encouraged sensing (*p* = 0.88) and RV capture issues were not seen more during Encouraged Pacing (*p* = 1). In 10 episodes, RV loss—of capture was unmasked by the Encouraged Pacing mode (example in Figure 3), but the inverse was also true in eight cases, where the anomaly was visible during the passive mode and not during Encouraged Pacing (example in Figure 4). While RV undersensing was unmasked in 13 cases by Encouraged Sensing (example in Figure 5), the inverse was also true in eight cases (Figure 6). For the LV lead, loss-of-capture anomalies were not more likely to be seen during Encouraged Pacing when compared with the passive mode (*p* = 1).

## 4. Discussion

### 4.1. Contribution of Active EGM

With an ever-increasing number of cardiac implantable electronic devices being implanted, remote monitoring teams are receiving a growing amount of data [25]. The incoming data flow may be split into three categories: scheduled transmissions at regular intervals, day-to-day alerts transmitted by the prosthesis in the event of a detected anomaly, and manual transmissions initiated by the patient. Each transmission contains a large amount of information, enabling assessment of the device operating status and the patient’s rhythm, with EGM tracings illustrating the anomalies detected. EGMs are essential as they confirm the diagnosis, which may (or may not) have been suspected by the device’s automatic measurements. Most of these transmissions are accompanied by a real-time EGM, which illustrates the real-time operation at the time of transmission. These EGMs are presented as a snapshot of the patient’s rhythm during transmission and have benefits that are not restricted to diagnosing lead anomalies. For example, when paroxysmal arrhythmia has been recorded, a real-time EGM allows the healthcare professional to evaluate whether the patient is still in arrhythmia. These EGMs can also be compared to earlier similar EGMs to evaluate whether a change has occurred. 

Our study revealed that the prevalence of abnormalities found on periodic EGMs from Biotronik pacemakers was 6.7% (i.e., one in 15 traces presented an abnormality). This prevalence was previously unknown and supports the value of systematically reading this type of EGM to reveal functional anomalies, most of which have no dedicated screening algorithm. We also show that the acquisition of additional EGMs enables the diagnosis of more anomalies than when compared to a passive EGM only. There are two possible explanations for this finding: (1) longer tracings (36 s versus 12 s) increase the chance of detecting intermittent anomalies, and (2) the temporary changes in atrioventricular delay and lower rate favor sensing and pacing, which unmask anomalies. In our study, we attempted to weigh both explanations through a comparison between the modes. Indeed, for atrial capture and noise related to atrial lead dysfunction, the active mode with Encouraged Pacing was associated with a higher yield. While the same holds true for far-field R-wave oversensing on the atrial lead, the clinical importance may be limited, as the anomaly could be mode-dependent. For the RV and LV lead, however, we were not able to show significant advantages of one active mode versus the passive mode in our study with over 2700 patients. The higher rate of anomalies found during the active mode was attributed to the longer EGM length. Our results show that a longer recording time is a major factor in improving the detection of intermittent anomalies.

### 4.2. Clinical Significance

**Figure 7:**
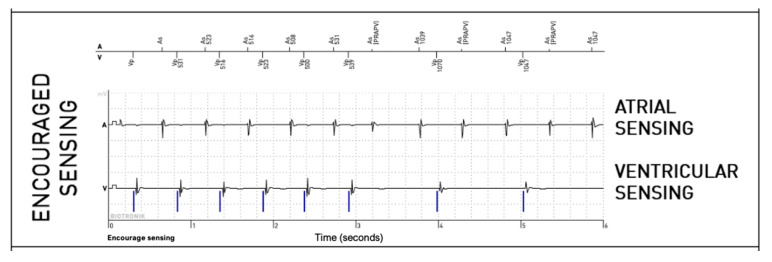
Example of a 2:1 block due to the extension of the AV interval by the “Encouraged Sensing” algorithm during sinus tachycardia in a patient with AV block.

Analysis of periodic EGMs is considered to be low-yield when compared to analysis of other types of registered and transmitted episodes for the detection of anomalies. However, we must keep in mind that none of the current devices are able to self-detect undersensing of the cardiac signals. Also beat-to-beat capture analysis is not always possible. One of the reasons for low yield of passive periodic EGMs is that it is challenging to evaluate, in real time, the quality of sensing (for a patient receiving 100% pacing) or the efficiency of capture (for a patient in spontaneous rhythm). This is why the concept of active EGM was proposed, with the goal to increasing the yield in finding anomalies in pacemakers through remote monitoring. The possibility of recording active EGMs with temporary modification of programming to increase diagnostic yield is a further step in the gradual evolution toward more remote follow-up and less face-to-face follow-up. While we expected to show that active modes increase the diagnostic yield, the results were limited to the atrial lead, with no significant gain for the RV and LV leads. We also showed the value of recording long periodic EGMs, as diagnostic yield doubled when the tracing was three times as long. We should also take into account that there are potential adverse events during the registration of active EGMs. These adverse events are expected as parameters are temporarily altered in order to encourage sensing or pacing. During Encouraged Sensing, the AV interval is extended (to 450 ms in DDD-ADI mode), which may induce pacemaker-mediated tachycardia but also inherently lowers the 2:1 rate by extension of the total atrial refractory period. An example of 2:1 block during sinus tachycardia is shown in Figure 7. While we have seen no examples in our study, Encouraged Pacing may, in theory, be arrhythmogenic through changes in cycle intervals, alterations in ventricular contraction, or the introduction of blanking periods resulting in functional undersensing. After the analysis of over 7000 EGMs in our study, we postulate that the adverse effects of active periodic EGMs are extremely rare. Limiting the algorithm of active EGMS to heart rates under 100 beats per minute would further diminish these risks. In the new generation of Biotronik pacemakers (Amvia Sky), active modes have been abolished, and the periodic EGM, entirely passive, has been extended to 30 s. This development is in line with our results, which indicate an interest in having EGMs longer than 12 s and limited value in active modes.

The relatively high incidence of abnormalities, which may modify management, seems to justify the systematic reading of long passive EGMs. On the other hand, we should not ignore the importance of the work burden imposed by correct remote monitoring, which may make it a victim of its own success [26]. To avoid a significant increase in the work burden of the medical and paramedical team carrying out the task of analyzing these tracings, it is clear that an automated screening tool would be the preferred solution for an exhaustive interpretation of this type of tracing [27]. A machine learning approach seems particularly promising in this context, as it could screen digital signals for anomalies on the remote monitoring platform. In a similar context, we have previously shown that multiple machine learning algorithms were able to distinguish noise from non-sustained ventricular arrhythmia in Boston Scientific pacemakers and implantable cardioverter defibrillators as well as an experienced remote monitoring team.

### 4.3. Study Limitations

There are important limitations to this study. As undersensing and loss of ventricular capture are rare events, the study may lack the power to demonstrate a significant difference for these specific issues between passive and active modes. However, a possible difference would be small, and the clinical implications would therefore be limited. The study focuses on pacemakers from a single implantable cardiac device manufacturer, and the application of the results to other manufacturers or device types remains to be shown. While the adverse events associated with the active modes appear limited and rare, we may have missed extremely rare events, and this may be different for other types of devices. We also did not investigate the clinical consequences of the anomalies. While our cardiac electrophysiology team is highly experienced in analyzing periodic electrograms, we acknowledge that some episodes might have been missed or falsely identified. However, we believe that any such errors would be evenly distributed across the modes (passive, Encouraged Sensing, and Encouraged Pacing) and therefore would not introduce a systematic bias into our findings.

## 5. Conclusions

The systematic review of periodic EGMs reveals abnormalities in 6.7% of cases, highlighting the importance of their routine analysis. Active registration modes significantly enhance diagnostic yield for atrial lead anomalies but show limited benefits for ventricular leads. Extended passive EGMs contribute substantially to anomaly detection, demonstrating the value of longer recording durations. The findings suggest that focusing on passive periodic EGMs of longer durations may reduce the need for active pacing adjustments. Further development of automated screening tools, potentially leveraging machine learning, could optimize the interpretation and reduce the burden of remote monitoring.

## Figures and Tables

**Table 1:** Number of anomalies detected during each mode, distribution across the modes, and comparison between normal mode and each active mode. RV = right ventricle. LV = left ventricle.

	Passive	Encour. Sensing	*p* vs. Passive	Encour. Pacing	*p* vs. Passive
*ATRIAL LEAD Anomalies*					
*Atrial undersensing (N = 66)*	47	54	0.48	36	0.08
*Atrial oversensing: far-field (N = 188)*	69	142	<0.001	23	<0.001
*Atrial oversensing: lead dysfunction (N = 29)*	5	11	0.5	20	0.02
*Atrial oversensing: external source (N = 2)*	1	1	-	2	-
*Loss of atrial capture (N = 74)*	27	18	0.5	59	<0.001
*RV LEAD Anomalies*					
*RV undersensing (N = 36)*	19	24	0.86	8	0.06
*RV oversensing: far-field (N = 2)*	1	2	-	1	-
*RV oversensing: lead dysfunction (N = 0)*	0	0	-	0	-
*RV oversensing: external source (N = 0)*	0	0	-	0	-
*Loss of RV capture (N = 29)*	19	1	<0.001	21	1
*LV LEAD Anomaly*					
*Loss of LV capture (N = 47)*	44	23	<0.001	42	1

## Data Availability

Data available for sharing after reasonable request.
